# Supplementary figures and images for: Knockout of the orphan membrane transporter Slc22a23 leads to a lean and hyperactive phenotype with a small hippocampal volume
Source: PLoS One. 2024 Aug 28;19(8):e0309461. doi: 10.1371/journal.pone.0309461 (PMC11356391; doi:10.1371/journal.pone.0309461)

**A**

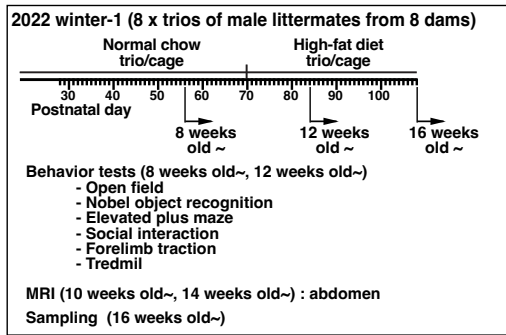

**B**

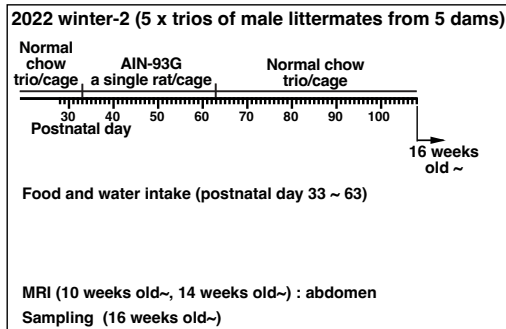

**C**

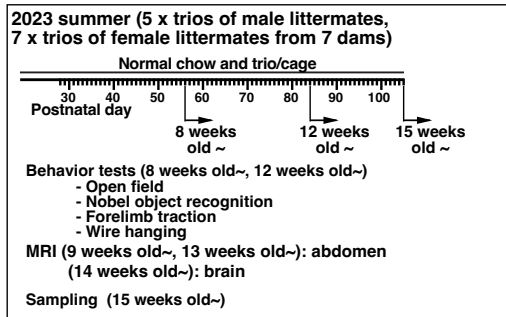

Supplement: S1 Fig — (A) 2022 winter-1, (B) 2022 winter-2, (C) 2023 summer. Limited resources were the primary reason for the choice of the sample size in this study [46] because we had limited breeding space available and had to use gender and litter-matched groups of Slc22a23+/+, Slc22a23+/−, Slc22a23−/− rats in the experiment. The expected effect size was not available before the experiment started because the phenotypic analysis of Slc22a23 knockout rats was novel. A post-hoc power analysis was performed using the statistical software G*Power (version 3.1) [47] with the following settings (Test family: F tests, Statistical test: ANOVA, Type of power analysis: Post hoc, Effect size: 0.5, alpha error probability: 0.05, Number of groups: 3). The calculated values of the power (1-beta error probability) were 0.31 (when the total sample size is 15), 0.46 (when the total sample size is 21) and 0.52 (when the total sample size is 24). The power would be 0.77 (total sample size 39) if we combined two datasets (e.g. the results of 8 weeks in 2022 winter-1 and 2023 summer). (PDF) [file pone.0309461.s001.pdf]

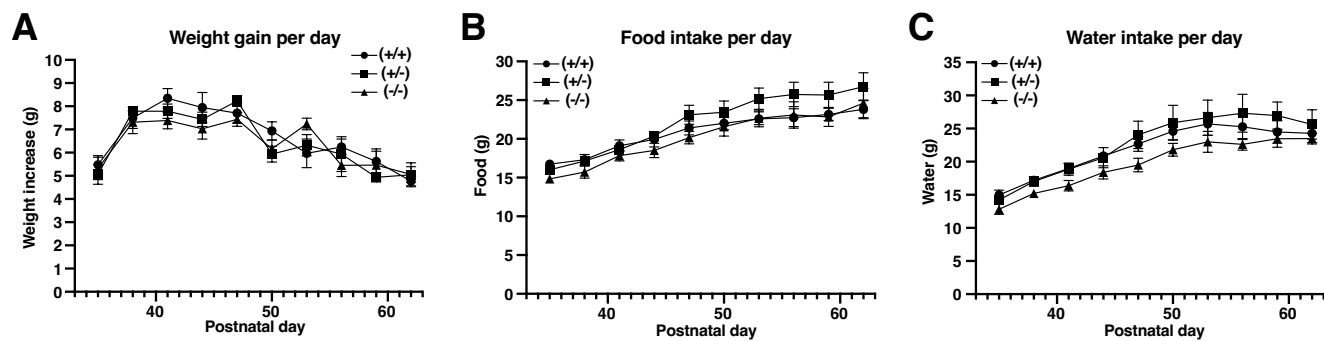

Supplement: S2 Fig — After being housed in a trio (Slc22a23+/+, Slc22a23+/−, and Slc22a23−/−), each rat was individually housed in a cage from postnatal day (P)33 to P63 to measure food and water intake. Their (A) body weight, (B) food intake, and (C) water intake were measured every 3 days. The winter-2022-2 results (male, n = 5 in each group) are shown. (PDF) [file pone.0309461.s002.pdf]

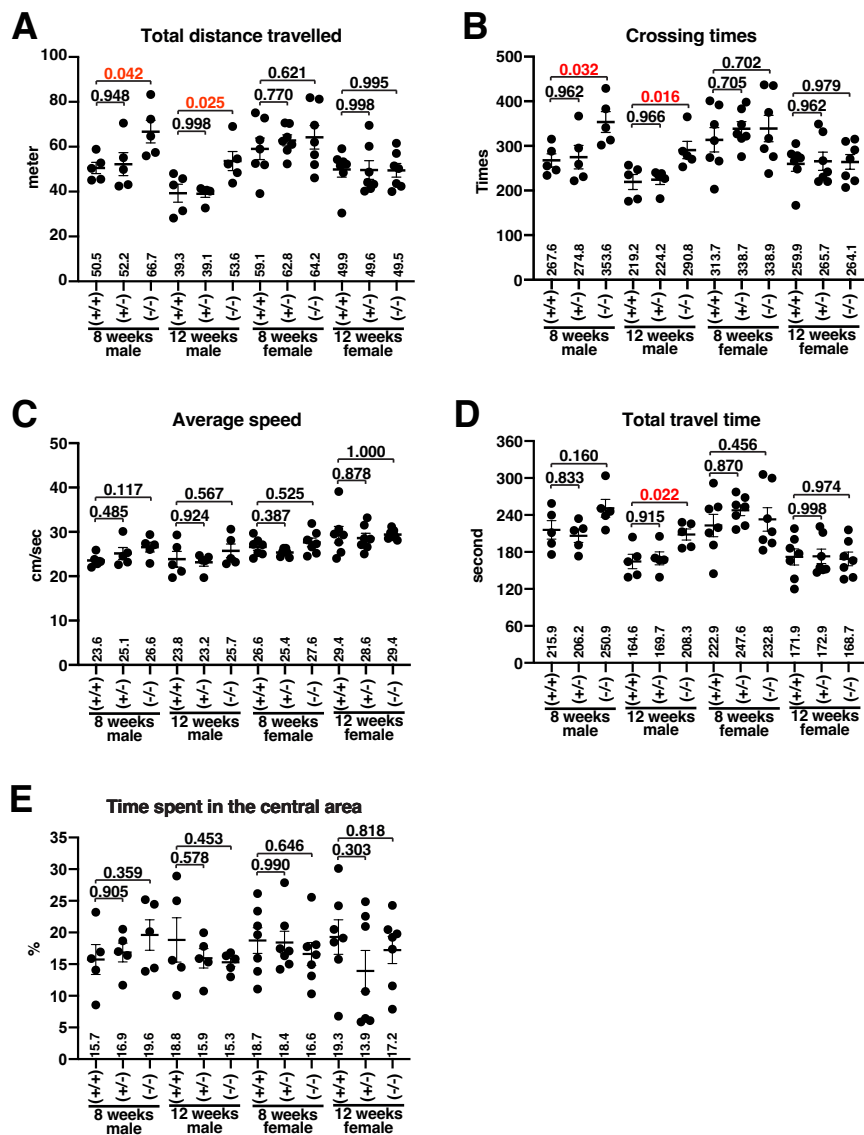

Supplement: S3 Fig — The results of the 2023 summer experiment (male: n = 5 in each group, female: n = 7 in each group) are shown as in Fig 5A. The factorial ANOVA of the data in S3 Fig can be found in Panel B of S2 Table. (PDF) [file pone.0309461.s003.pdf]

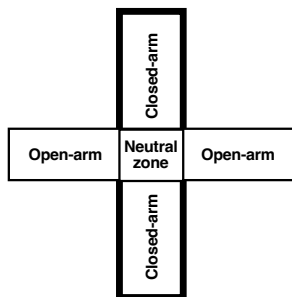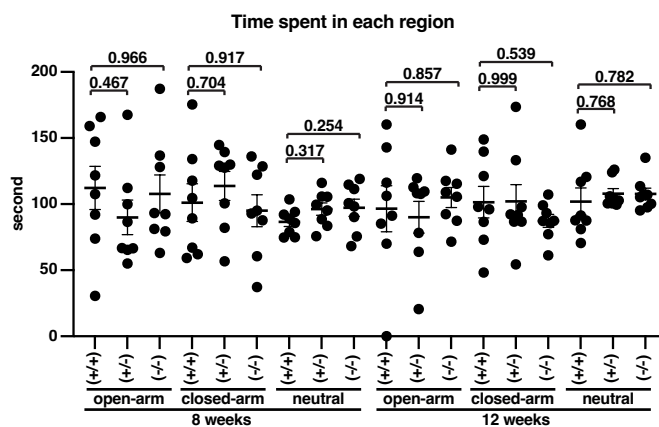

Supplement: S4 Fig — The apparatus had four arms (50 cm length, 10 cm width, 51.5 cm height); two of the arms lacked walls (open arms) and the other two arms had walls (closed arms). At the start of the test, the test rat was placed in the central zone of the apparatus. The movement of the test rat was recorded using a CompACT video tracking system (Muromachi Kikai) for 5 minutes. The width of the border was set to 5 cm. The time spent in each arm and the number of entries into each arm were recorded. The 2022 winter-1 results (male: n = 8 in each group) are shown. The error bars show the mean ± SEM. The p-values were calculated using Dunnett’s multiple comparisons with control +/+ rats. The factorial ANOVA of the data in S4 Fig can be found in Panel C of S2 Table. (PDF) [file pone.0309461.s004.pdf]

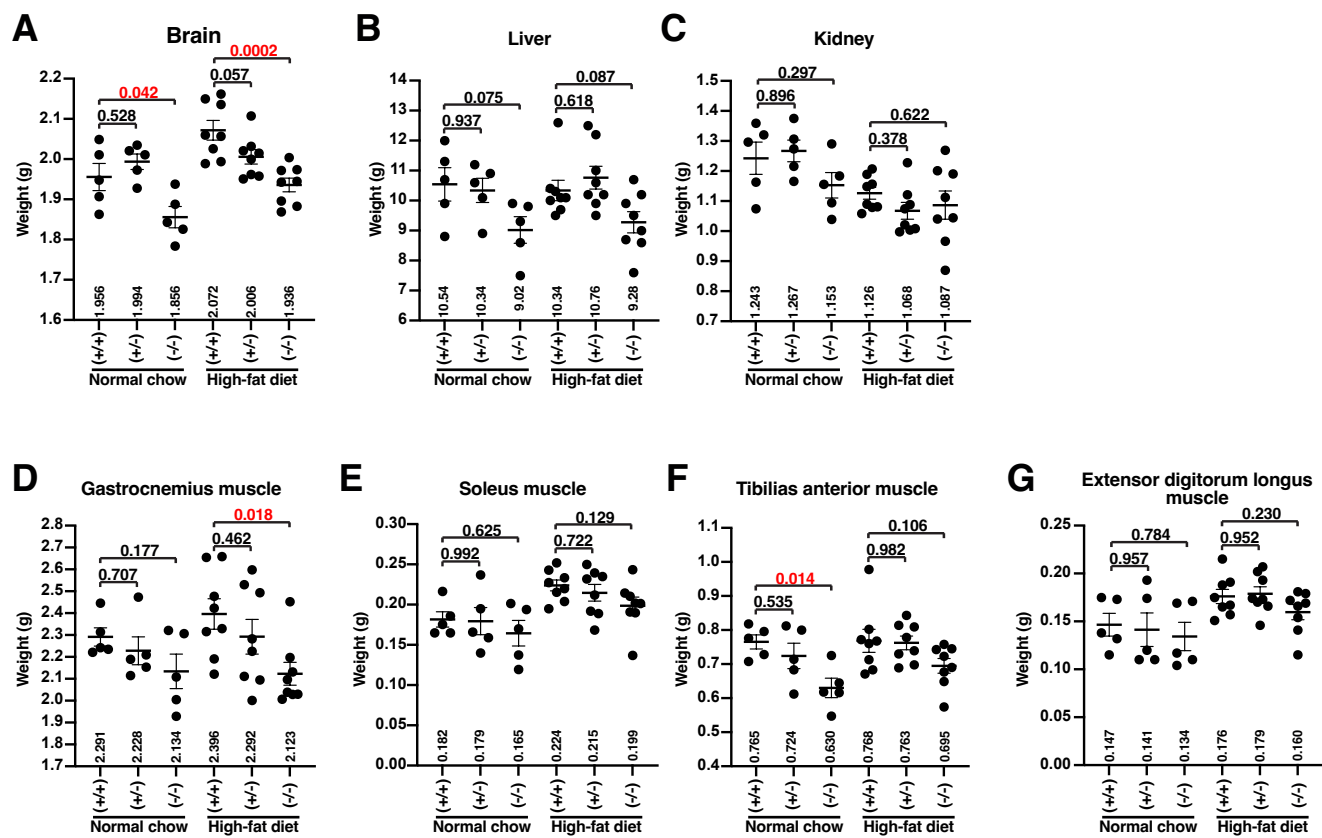

Supplement: S5 Fig — The results are shown as in Fig 8D, but the organs were isolated from 2022 winter-1 (male, n = 8, high-fat diet) and 2022 winter-2 (male, n = 5, normal chow) animals at 16 weeks of age. The error bars show the mean ± SEM. The p-values were calculated using Dunnett’s multiple comparisons with control +/+ rats. The factorial ANOVA of the data in S5 Fig can be found in Panel E of S3 Table. (PDF) [file pone.0309461.s005.pdf]
