# Supplementary material for: Knockout of the orphan membrane transporter Slc22a23 leads to a lean and hyperactive phenotype with a small hippocampal volume
Source: PLoS One. 2024 Aug 28;19(8):e0309461. doi: 10.1371/journal.pone.0309461 (PMC11356391; doi:10.1371/journal.pone.0309461)
Supplement: S2 Table — Values below each genotype are mean ± SEM. Factorial ANOVA (with repeated measurements) was performed with (A) ‘Genotype’ as a between-subjects factor, and ‘Age’ as a within-subjects factor; (B) ‘Genotype’ and ‘Gender’ as between-subjects factors, and ‘Age’ as a within-subjects factor; (C) ‘Genotype’ as a between-subjects factor, and ‘Age’ as a within-subjects factor; (D) ‘Genotype’ as a between-subjects factor, and ‘Age’ and ‘Day’ as within-subjects factors; (E) ‘Genotype’ as a between-subjects factor, and ‘Age’ and ‘Intruder’ as within-subjects factors. (PDF) [file pone.0309461.s007.pdf]

A

		(+/+)	(+/-)	(-/-)		Genotype	Age	Genotype x Age
Open field test: total distance travelled (meter)								
(Fig 5 A1)	8 weeks (male)	57.40 ± 2.73	62.98 ± 3.69	76.73 ± 6.24	DF	2	1	2
	12 weeks (male)	64.85 ± 4.24	72.09 ± 4.62	92.28 ± 5.23	F ratio	13.8351	8.141	0.4329
					Prob > F	< 0.0001	0.0110	0.6555
Open field test: crossing times (times)								
(Fig 5 A2)	8 weeks (male)	294.50 ± 13.17	316.63 ± 21.44	384.13 ± 31.81	DF	2	1	2
	12 weeks (male)	320.63 ± 18.16	359.88 ± 22.87	448.50 ± 25.61	F ratio	11.9440	5.6639	0.3487
					Prob > F	0.0003	0.0274	0.7098
Open field test: average speed (cm/sec)								
(Fig 5 A3)	8 weeks (male)	25.28 ± 0.76	26.54 ± 1.08	29.90 ± 1.75	DF	2	1	2
	12 weeks (male)	29.58 ± 1.66	29.74 ± 1.45	34.40 ± 1.74	F ratio	6.1443	11.3627	0.1160
					Prob > F	0.0049	0.0220	0.8930
Open field test: total travel time (second)								
(Fig 5 A4)	8 weeks (male)	229.90 ± 11.35	236.55 ± 7.99	259.14 ± 12.07	DF	2	1	2
	12 weeks (male)	220.78 ± 12.67	242.04 ± 9.21	268.44 ± 8.08	F ratio	6.5529	0.0532	0.4711
					Prob > F	0.0059	0.8198	0.6310
Open field test: time spent in the central area (%)								
(Fig 5 A5)	8 weeks (male)	19.96 ± 4.64	18.54 ± 3.48	16.91 ± 1.41	DF	2	1	2
	12 weeks (male)	11.09 ± 2.12	14.21 ± 1.52	12.34 ± 1.46	F ratio	0.2150	6.7614	0.4051
					Prob > F	0.8083	0.0167	0.6720

B

		(+/+)	(+/-)	(-/-)		Genotype	Gender	Age	Genotype x Gender	Genotype x Age	Gender x Age	Genotype x Gender x Age
Open field test: total distance travelled (meter)												
(S3 Fig A)	8 weeks (male)	50.54 ± 2.53	52.22 ± 5.14	66.74 ± 5.05	DF	2	1	1	2	2	1	2
	12 weeks (male)	39.31 ± 3.98	39.07 ± 1.61	53.64 ± 4.27	F ratio	3.7586	3.8896	48.8064	2.4897	0.4078	0.0015	0.0901
	8 weeks (female)	59.08 ± 4.76	62.82 ± 2.44	64.20 ± 5.28	Prob > F	0.0349	0.0579	< 0.0001	0.0999	0.6687	0.9691	0.9141
	12 weeks (female)	49.92 ± 3.47	49.63 ± 4.19	49.48 ± 3.02								
Open field test: crossing times (times)												
(S3 Fig B)	8 weeks (male)	267.6 ± 14.86	274.8 ± 26.33	353.6 ± 23.47	DF	2	1	1	2	2	1	2
	12 weeks (male)	219.2 ± 17.00	224.2 ± 10.78	290.8 ± 19.41	F ratio	3.6973	2.9214	38.7034	2.4624	0.2773	0.4636	0.0643
	8 weeks (female)	313.71 ± 27.18	338.71 ± 15.72	338.86 ± 29.60	Prob > F	0.0365	0.0976	< 0.0001	0.1020	0.7598	0.5012	0.9379
	12 weeks (female)	259.86 ± 16.78	265.71 ± 20.32	264.14 ± 16.52								
Open field test: average speed (cm/sec)												
(S3 Fig C)	8 weeks (male)	23.56 ± 0.66	25.14 ± 1.33	26.56 ± 1.07	DF	2	1	1	2	2	1	2
	12 weeks (male)	23.84 ± 1.78	23.16 ± 0.89	25.74 ± 1.54	F ratio	2.2464	18.7515	2.2681	0.6310	0.3089	8.6876	0.5984
	8 weeks (female)	26.62 ± 0.74	25.36 ± 0.31	27.63 ± 0.97	Prob > F	0.1230	0.0001	0.1427	0.5389	0.7366	0.0062	0.5562
	12 weeks (female)	29.37 ± 1.85	28.61 ± 1.08	29.4 ± 0.41								
Open field test: total travel time (second)												
(S3 Fig D)	8 weeks (male)	215.92 ± 15.01	206.18 ± 10.72	250.94 ± 14.50	DF	2	1	1	2	2	1	2
	12 weeks (male)	164.56 ± 11.70	169.74 ± 10.53	208.28 ± 8.96	F ratio	1.7399	0.0005	83.5385	2.4265	0.0477	2.8657	0.9191
	8 weeks (female)	222.9 ± 18.02	247.61 ± 8.58	232.81 ± 19.04	Prob > F	0.1928	0.9829	< 0.0001	0.1055	0.9535	0.1009	0.4098
	12 weeks (female)	171.94 ± 13.09	172.9 ± 11.69	168.71 ± 11.14								
Open field test: time spent in the central area (%)												
(S3 Fig E)	8 weeks (male)	15.73 ± 2.36	16.85 ± 1.48	19.61 ± 2.40	DF	2	1	1	2	2	1	2
	12 weeks (male)	18.82 ± 3.50	15.94 ± 1.53	15.28 ± 0.69	F ratio	0.3990	0.0372	0.9848	0.1729	2.2941	0.0470	2.1433
	8 weeks (female)	18.73 ± 2.03	18.42 ± 1.78	16.61 ± 1.81	Prob > F	0.6745	0.8483	0.3289	0.8421	0.1183	0.8298	0.1349
	12 weeks (female)	19.28 ± 2.73	13.92 ± 3.23	17.23 ± 2.13								

C

		(+/+)	(+/-)	(-/-)		Genotype	Age	Genotype x Age
Elevated plus-maze: time spent in the open-arm (second)								
(S4 Fig)	8 weeks (male)	112.23 ± 16.34	89.99 ± 13.04	107.69 ± 14.34	DF	2	1	2
	12 weeks (male)	96.53 ± 17.36	90.06 ± 11.99	105.03 ± 7.54	F ratio	0.4943	1.1035	0.7034
					Prob > F	0.6164	0.3063	0.5071
Novel object recognition test: discrimination ratios								
(Fig 5 B2)	8 weeks (male)	-0.030 ± 0.057	0.142 ± 0.036	0.129 ± 0.039	DF	2	1	2
	12 weeks (male)	0.005 ± 0.057	-0.098 ± 0.028	0.176 ± 0.040	F ratio	6.1179	1.9947	6.8904
					Prob > F	0.0093	0.1751	0.0062

D

		(+/+)	(+/-)	(-/-)		Genotype	Age	Day	Genotype x Age	Genotype x Day	Age x Day	Genotype x Age x Day
Social interaction test: discrimination ratios of rat A on day 2 and day 3												
(Fig 6 B)	8 weeks on Day 2	0.367 ± 0.129	0.383 ± 0.097	0.426 ± 0.046	DF	2	1	1	2	2	1	2
	8 weeks on Day 3	0.191 ± 0.110	0.180 ± 0.083	0.306 ± 0.064	F ratio	0.7755	17.9974	1.7332	0.5782	0.1886	12.7785	1.1903
	12 weeks on Day 2	0.533 ± 0.081	0.371 ± 0.120	0.572 ± 0.061	Prob > F	0.4734	0.0004	0.2023	0.5697	0.8295	0.0018	0.3239
	12 weeks on Day 3	0.630 ± 0.060	0.533 ± 0.045	0.558 ± 0.049								

E

		(+/+)	(+/-)	(-/-)		Genotype	Age	Intruder	Genotype x Age	Genotype x Intruder	Age x Intruder	Genotype x Age x Intruder
Social interaction test: discrimination ratios of rat A and rat B on day 3												
(Fig 6 C)	8 weeks with rat A	0.191 ± 0.110	0.180 ± 0.083	0.306 ± 0.064	DF	2	1	1	2	2	1	2
	8 weeks with rat B	0.185 ± 0.072	0.072 ± 0.066	0.091 ± 0.080	F ratio	1.1215	38.4185	5.5433	0.3545	0.9004	0.0827	0.7357
	12 weeks with rat A’	0.630 ± 0.060	0.533 ± 0.045	0.558 ± 0.049	Prob > F	0.3453	< 0.0001	0.0284	0.7057	0.4216	0.7765	0.4910
	12 weeks with rat B’	0.600 ± 0.089	0.393 ± 0.128	0.456 ± 0.109								

Uchimura et al. S2 Table
