# Supplementary material for: Knockout of the orphan membrane transporter Slc22a23 leads to a lean and hyperactive phenotype with a small hippocampal volume
Source: PLoS One. 2024 Aug 28;19(8):e0309461. doi: 10.1371/journal.pone.0309461 (PMC11356391; doi:10.1371/journal.pone.0309461)
Supplement: S3 Table — Values below each genotype are mean ± SEM. Factorial ANOVA (with repeated measurements) was performed with (A) ‘Genotype’ as a between-subjects factor, and ‘Age’ as a within-subjects factor; (B) ‘Genotype’ and ‘Gender’ as between-subjects factors, and ‘Age’ as a within-subjects factor; (C) ‘Genotype’ as a between-subjects factor, and ‘Age’ as a within-subjects factor. In (D) and (E), factorial ANOVA (without repeated measurements) was performed. (PDF) [file pone.0309461.s008.pdf]

A

		(+/+)	(+/-)	(-/-)		Genotype	Age	Genotype x Age
Forelimb traction (kg) (Fig 7A)	8 weeks (male)	0.583 ± 0.074	0.511 ± 0.089	0.552 ± 0.098	DF	2	1	2
	12 weeks (male)	0.656 ± 0.045	0.605 ± 0.036	0.529 ± 0.036	F ratio	0.8512	0.8621	0.1744
					Prob > F	0.4381	0.3678	0.8416

B

		(+/+)	(+/-)	(-/-)		Genotype	Gender	Age	Genotype x Gender	Genotype x Age	Gender x Age	Genotype x Gender x Age
Forelimb traction (kg) (Fig 7B)	8 weeks (male)	0.666 ± 0.077	0.615 ± 0.062	0.654 ± 0.028	DF	2	1	1	2	2	1	2
	12 weeks (male)	0.679 ± 0.025	0.674 ± 0.038	0.704 ± 0.029	F ratio	0.3480	0.4558	3.5648	0.3129	1.1050	0.1008	0.3495
	8 weeks (female)	0.633 ± 0.049	0.582 ± 0.025	0.643 ± 0.051	Prob > F	0.7089	0.5048	0.0687	0.7337	0.3443	0.7531	0.7078
	12 weeks (female)	0.626 ± 0.034	0.719 ± 0.055	0.686 ± 0.013								

C

		(+/+)	(+/-)	(-/-)		Genotype	Age	Genotype x Age
Treadmill (meter) (Fig 7C)	8 weeks (male)	602.86 ± 142.37	256.99 ± 106.88	1034.75 ± 442.12	DF	2	1	2
	12 weeks (male)	667.34 ± 220.40	291.79 ± 103.97	2180.88 ± 741.90	F ratio	8.4976	1.5006	1.1639
					Prob > F	0.0016	0.2370	0.3355

D

		(+/+)	(+/-)	(-/-)		Genotype	Gender	Genotype x Gender
Four-limb hanging (second) (Fig 7D)	12 weeks (male)	9.47 ± 2.39	22.00 ± 13.40	68.83 ± 17.28	DF	2	1	2
	12 weeks (female)	99.02 ± 19.88	163.95 ± 26.63	240.88 ± 92.67	F ratio	2.1629	11.3989	0.3660
					Prob > F	0.1326	0.0020	0.6965
Holding impulse (Fig 7E)	12 weeks (male)	3494.57 ± 891.19	7757.42 ± 4527.15	22941.79 ± 5970.65	DF	2	1	2
	12 weeks (female)	23359.53 ± 4903.38	38360.32 ± 5895.56	51874.42 ± 19644.77	F ratio	2.5720	9.2789	0.1474
					Prob > F	0.0931	0.0048	0.8636

E

		(+/+)	(+/-)	(-/-)		Genotype	Food	Genotype x Food
Brain (g) (S5 Fig A)	Normal chow	1.956 ± 0.034	1.994 ± 0.019	1.856 ± 0.026	DF	2	1	2
	High-fat diet	2.072 ± 0.025	2.006 ± 0.018	1.936 ± 0.017	F ratio	14.9250	13.0018	2.5068
					Prob > F	< 0.0001	0.0010	0.0969
Liver (g) (S5 Fig B)	Normal chow	10.54 ± 0.556	10.34 ± 0.403	9.02 ± 0.444	DF	2	1	2
	High-fat diet	10.338 ± 0.340	10.763 ± 0.381	9.275 ± 0.353	F ratio	7.0675	0.2184	0.3040
					Prob > F	0.0028	0.6433	0.7399
Kidney (g) (S5 Fig C)	Normal chow	1.243 ± 0.053	1.267 ± 0.036	1.153 ± 0.043	DF	2	1	2
	High-fat diet	1.126 ± 0.020	1.068 ± 0.028	1.087 ± 0.047	F ratio	1.4837	16.0184	1.4929
					Prob > F	0.2415	0.0003	0.2395
Gastrocnemius muscle (g) (S5 Fig D)	Normal chow	2.291 ± 0.042	2.228 ± 0.063	2.134 ± 0.079	DF	2	1	2
	High-fat diet	2.396 ± 0.070	2.292 ± 0.079	2.123 ± 0.052	F ratio	4.7250	0.8314	0.3462
					Prob > F	0.0157	0.3685	0.7099
Soleus muscle (g) (S5 Fig E)	Normal chow	0.182 ± 0.010	0.179 ± 0.017	0.165 ± 0.016	DF	2	1	2
	High-fat diet	0.224 ± 0.007	0.215 ± 0.010	0.199 ± 0.011	F ratio	1.7672	15.4538	0.0690
					Prob > F	0.1866	0.0004	0.9334
Tibilas anterior muscle (g) (S5 Fig F)	Normal chow	0.765 ± 0.021	0.724 ± 0.037	0.630 ± 0.029	DF	2	1	2
	High-fat diet	0.768 ± 0.034	0.763 ± 0.020	0.695 ± 0.022	F ratio	7.2666	2.2810	0.5781
					Prob > F	0.0024	0.1405	0.5665
Extensor digitorum longus muscle (g) (S5 Fig G)	Normal chow	0.147 ± 0.012	0.141 ± 0.018	0.134 ± 0.015	DF	2	1	2
	High-fat diet	0.176 ± 0.007	0.179 ± 0.007	0.160 ± 0.008	F ratio	1.1449	12.6950	0.1673
					Prob > F	0.3306	0.0011	0.8466

Uchimura et al. S3 Table
