# Supplementary material for: Knockout of the orphan membrane transporter Slc22a23 leads to a lean and hyperactive phenotype with a small hippocampal volume
Source: PLoS One. 2024 Aug 28;19(8):e0309461. doi: 10.1371/journal.pone.0309461 (PMC11356391; doi:10.1371/journal.pone.0309461)
Supplement: S1 Raw images — (PDF) [file pone.0309461.s009.pdf]

Fig 2B: gel image

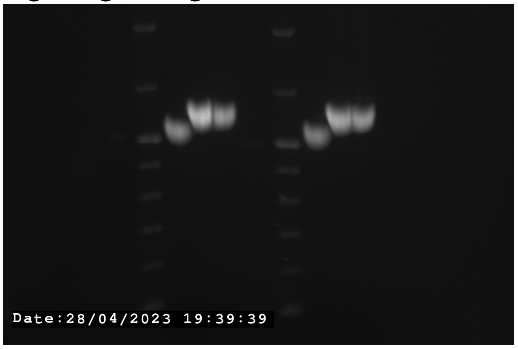

Fig 3B: gel image

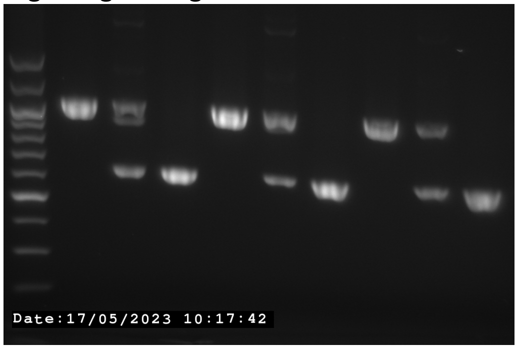

Fig 3D: blot image
(anti-SLC22A23)

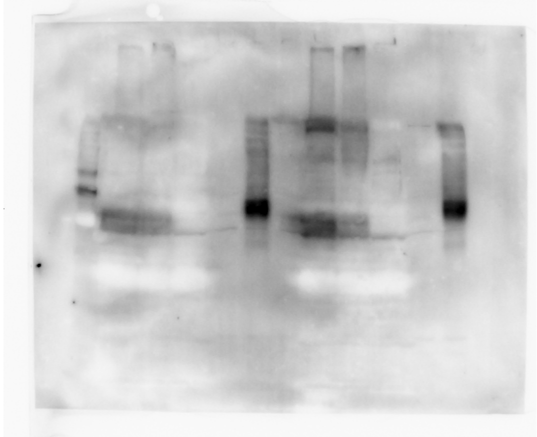

Fig 3D: blot image
(anti-HA)

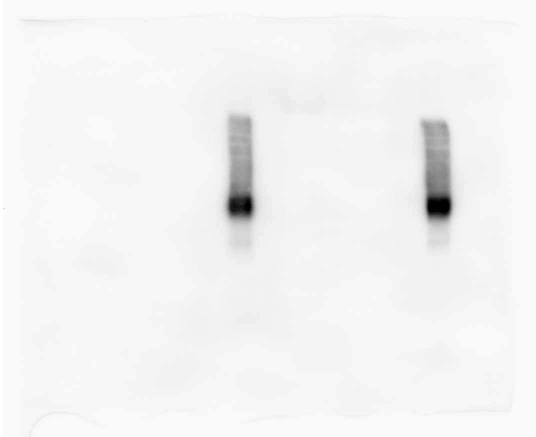

Fig 3D: blot image
(anti-Tubulin beta III)

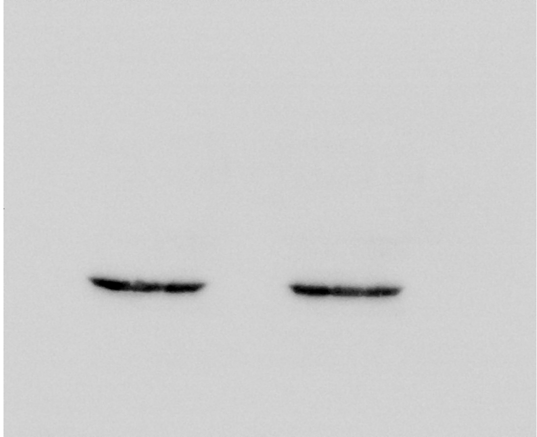

Fig 3D: blot image
(anti-GFAP)

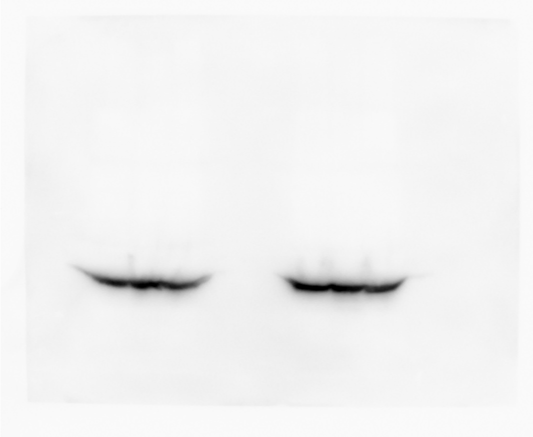

Fig 3D: blot image
(anti-CNPase)

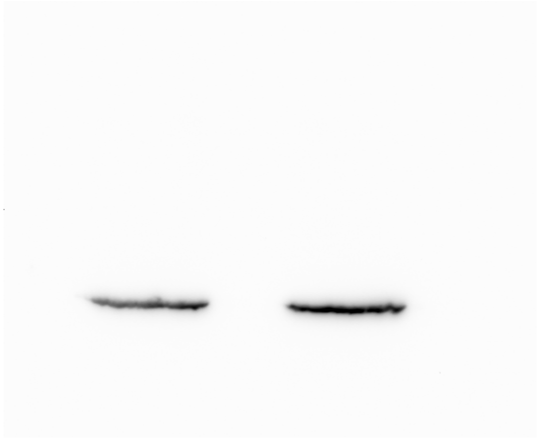

Uchimura et al. S1_raw_images
