# Supplementary material for: Knockout of the orphan membrane transporter Slc22a23 leads to a lean and hyperactive phenotype with a small hippocampal volume
Source: PLoS One. 2024 Aug 28;19(8):e0309461. doi: 10.1371/journal.pone.0309461 (PMC11356391; doi:10.1371/journal.pone.0309461)
Supplement: Attachment — Submitted filename: Response to Reviewers (Uchimura).pdf [file pone.0309461.s011.pdf]

Response to Reviewers

Yasuhiro Uchimura, PhD
Division of Anatomy and Cell Biology
Department of Anatomy
Shiga University of Medical Science
Seta Tsukinowa-cho, Otsu, Shiga, Japan 520-2192

July 29, 2024

Dear Professor Nemoto and the reviewers,

Thank you very much for reviewing my manuscript. Here I respond to each point raised by the academic editor and the reviewers.

Journal Requirements:

1. When submitting your revision, we need you to address these additional requirements.

Please ensure that your manuscript meets PLOS ONE's style requirements, including those for file naming. The PLOS ONE style templates can be found at https://journals.plos.org/plosone/s/file?id=wjVg/PLOSOne_formatting_sample_main_body.pdf and https://journals.plos.org/plosone/s/file?id=ba62/PLOSOne_formatting_sample_title_authors_affiliations.pdf

I checked the links and followed the instructions.

2. To comply with PLOS ONE submissions requirements, in your Methods section, please provide additional information regarding the experiments involving animals and ensure you have included details on (a) methods of sacrifice, (b) methods of anesthesia and/or analgesia, and (c) efforts to alleviate suffering.

I added these statements in the Method section of the manuscript.

3. Thank you for stating in your Funding Statement:
This work was supported by Japan Society for the Promotion of Science (JSPS; https://www.jsps.go.jp/) - JP 19K08274 [YU], JP 16H06277 / JP 22H04923 [YD], and JP 16H06276 [TM]. The funders had no role in the study design, data collection and analysis, decision to publish, or manuscript preparation.

Please provide an amended statement that declares all the funding or sources of support (whether external or internal to your organization) received during this study, as detailed online in our guide for authors at http://journals.plos.org/plosone/s/submit-now. Please also include the statement "There was no additional external funding received for this study." in your updated

Funding Statement.
Please include your amended Funding Statement within your cover letter. We will change the online submission form on your behalf.

I added the statement: "There was no additional external funding received for this study."

4. Your ethics statement should only appear in the Methods section of your manuscript. If your ethics statement is written in any section besides the Methods, please delete it from any other section.

I checked that my ethics statement only appears in the Methods section of my manuscript.

5. PLOS ONE now requires that authors provide the original uncropped and unadjusted images underlying all blot or gel results reported in a submission's figures or Supporting Information files. This policy and the journal's other requirements for blot/gel reporting and figure preparation are described in detail at https://journals.plos.org/plosone/s/figures#loc-blot-and-gel-reporting-requirements and https://journals.plos.org/plosone/s/figures#loc-preparing-figures-from-image-files. When you submit your revised manuscript, please ensure that your figures adhere fully to these guidelines and provide the original underlying images for all blot or gel data reported in your submission. See the following link for instructions on providing the original image data: https://journals.plos.org/plosone/s/figures#loc-original-images-for-blots-and-gels.
In your cover letter, please note whether your blot/gel image data are in Supporting Information or posted at a public data repository, provide the repository URL if relevant, and provide specific details as to which raw blot/gel images, if any, are not available. Email us at plosone@plos.org if you have any questions.

I added all blot/gel image data as S1_raw_images in the Supporting information.

Reviewer #1: *This study shows that uplegulation of Slc22a23 mRNA in the rat brain after undernutrition during fetal development. Then, the authors generated Slc22a23 knockout rats and found the KO rats showed lean, hyperactive, and reduced hippocampal volume. These results are interesting. I have some requests.*

1. If transcript of a Slc22a23 gene is important after undernutrition during fetal development, overexpression of Slc22a23 should have critical effects to pathophysiology. The authors should show this evidence, or at least discuss about it.

Thank you very much for pointing this out. We have a section entitled "Overexpression of the Slc22a23 gene" in the DISCUSSION section.

2. The authors should describe all F values, degrees of freedom, exact p values of main effects of genotype, sex, or age, and their interactions after two- or three-way ANOVA with repeated measure in supplementary table.

Thank you very much for pointing this out. We have added these data to the S2 and S3 Tables.

3. The authors should show representable MRI 3D images of KO and WT rat brains.

Thank you very much for pointing this out. We have added the 3D images of MRI to the Fig 9. I realised my mistake of using the wrong scale factor in the initial analysis of Stereo Investigator Fig 8F. I corrected the value in Fig 8F.

4. 'Written consent' is not necessary.

I removed them from the manuscript.

Reviewer #2: *Dear author;*
Your work has been prepared with a very original and sufficient methodology.
As a suggestion, the concentrations of antibodies examined in immunoblotting can also be presented.

I added the concentrations of antibodies in the sections of MATERIALS AND METHODS.

In addition, it would be useful to clearly state how the bands in the membranes obtained for western blot were calculated and which statistical method was used.
Best regards

I added the value in Fig3D and then added the following sentences in the sections of MATERIALS AND METHODS; The signals on the membrane were detected using a chemiluminescence imaging system (FUSION SOLO.6S.EDGE; Viber-Lourmat, Collégien, France) and saved in TIF format. The TIF files were opened in ImageJ (version 1.54g; NIH, US). The band intensities of the SLC22A23 protein, indicated by the arrowhead, were measured using the Analyze function in ImageJ. The band intensities of the SLC22A23 protein were compensated by the band intensities of the loading controls (anti-Tubulin betaIII, anti-GFAP or anti-CNPase). The relative band intensities of the SLC22A23 protein compared to +/+ are shown in Fig 3D.

Thank you very much for your consideration of this manuscript.

Yours sincerely,

Yasuhiro Uchimura
