# Supplementary material for: Knockout of the orphan membrane transporter Slc22a23 leads to a lean and hyperactive phenotype with a small hippocampal volume
Source: PLoS One. 2024 Aug 28;19(8):e0309461. doi: 10.1371/journal.pone.0309461 (PMC11356391; doi:10.1371/journal.pone.0309461)
Supplement: Attachment — Submitted filename: Response to Reviewers (Uchimura, 8AUG24).pdf [file pone.0309461.s012.pdf]

Response to Reviewers

Yasuhiro Uchimura, PhD
Division of Anatomy and Cell Biology
Department of Anatomy
Shiga University of Medical Science
Seta Tsukinowa-cho, Otsu, Shiga, Japan 520-2192

August 8, 2024

Dear Professor Nemoto and the reviewers,

Thank you very much for reviewing my manuscript. Here I respond to the point raised by reviewer #1.

Comments to the Author

1. If the authors have adequately addressed your comments raised in a previous round of review and you feel that this manuscript is now acceptable for publication, you may indicate that here to bypass the "Comments to the Author" section, enter your conflict of interest statement in the "Confidential to Editor" section, and submit your "Accept" recommendation.

Reviewer #1: (No Response)

Reviewer #2: All comments have been addressed

I addressed the comment received from Reviewer #1. Please see below.

6. Review Comments to the Author
Reviewer #1: The revised manuscript is improved following the reviewers' comments. One point should be addressed: Regarding the pathophysiology of overexpression of Slc22a23 gene, the authors should describe the limitation of this study using knockout rats instead of transgenic rats.

Thank you very much for pointing this out. I added the following sentence in the Discussion: However, the current study is limited in elucidating the pathophysiology of the Slc22a23 gene overexpression because Slc22a23 knockout rats were used instead of Slc22a23 overexpressing transgenic rats in this study.

Thank you very much for your consideration of this manuscript.

Yours sincerely,

Yasuhiro Uchimura
